# Illuminating Glucose: How to Unveil Organ‐Specific Insulin Resistance and Guide Metabolic Strategies in Diabetes

**DOI:** 10.1002/dmrr.70162

**Published:** 2026-03-25

**Authors:** Shawn Gugliandolo, Cassandra Morciano, Lucia Leccisotti, Umberto Capece, Gianfranco Di Giuseppe, Teresa Mezza, Gea Ciccarelli, Laura Soldovieri, Michela Brunetti, Adriana Avolio, Amelia Splendore, Alfredo Pontecorvi, Andrea Giaccari, Francesca Cinti

**Affiliations:** ^1^ Centro Malattie Endocrine e Metaboliche Dipartimento di Scienze Mediche e Chirurgiche Fondazione Policlinico Universitario A. Gemelli IRCCS and Dipartimento di Medicina e Chirurgia Traslazionale Università Cattolica del Sacro Cuore Rome Italy; ^2^ Medicina Nucleare Dipartimento di Diagnostica per Immagini e Radioterapia Oncologica Fondazione Policlinico Universitario A. Gemelli IRCCS and Università Cattolica del Sacro Cuore Rome Italy

**Keywords:** diabetes, insulin resistance, metabolism, PET‐CT, precision medicine, SGLT‐2i, [18F]‐FDG

## Abstract

Recent evidence has shown that muscle insulin resistance is not the only factor contributing to type 2 diabetes (T2D). Organ‐specific insulin resistance is increasingly recognised as a significant contributor to the metabolic changes that lead to hyperglycemia, although the precise extent of its impact remains unclear. The qualitative and quantitative aspects of regional insulin‐resistance in determining whole body insulin resistance and glucose uptake can be explored through positron emission tomography (PET) combined with computerised tomography images, using specific radio tracers like 2‐deoxy‐2‐[18F]fluoro‐D‐glucose ([18F]FDG). This approach provides new insight into organ‐specific glucose uptake allowing the visualisation of glucose metabolism. This review article seeks to highlight key findings from dynamic imaging, in terms of glucose uptake, focussing on the specific compartments (muscle, liver, adipose organ, heart, kidney and brain) in different metabolic conditions, such as insulin resistance and T2D, and during metabolic treatment. In essence, mapping these distinct organ contributions in the orchestra of glucose metabolism is forging a new frontier in personalised diabetes management, allowing for treatments uniquely tailored to individual metabolic needs.

**Abbreviations**
[18F]‐FDG: 2‐deoxy‐2‐[18F]fluoro‐D‐glucose
BAT: brown adipose tissue
BGU: brain glucose uptake
CT: computed tomography
FFA: free fatty acids
GIR: glucose infusion rate
GLUT: glucose transporters
HEC: euglycemic hyperinsulinemic clamp
HGP: hepatic glucose production
HGU: hepatic glucose uptake
MBF: myocardial blood flow
MBG: myocardial glucose uptake
MR: magnetic resonance
PET: positron emission tomography
SGLT: sodium‐glucose cotransporter
SGLT‐2i: sodium‐glucose cotransporter‐2 inhibitors
T2D: type 2 diabetes
WAT: White adipose tissue

## 1 Introduction

Type 2 diabetes (T2D) is a heterogenous group of disorders characterised by chronic hyperglycemia [1] resulting from multiple pathophysiological defects. The pancreatic beta cells respond to the increment in plasma glucose by increasing plasma insulin levels to maintain normal glucose tolerance, especially when there is an underlying insulin‐resistance [2, 3]. In insulin‐resistance, and T2D, glucose oxidation is impaired [4] along with the ability to shift substrate utilisation in favour of fatty acid oxidation over carbohydrates, which are unavailable as an energy source [5, 6, 7]. Therefore, gluco and lipotoxicity arise in the muscle, liver, adipocytes and beta cells contributing to insulin resistance and beta cell dysfunction in T2D [8].

Studies over the years have clarified that one of the hallmarks of diabetes is muscular insulin resistance manifesting as impaired glucose uptake [9]; however, whole body insulin resistance is not solely defined by muscular insulin resistance, even though the latter is certainly, both quantitatively and qualitatively, prominent. Under fasting conditions, when insulin levels are low, glucose uptake primarily occurs in insulin‐independent sites, including the brain and neural tissues (accounting for 50% of total glucose disposal), the splanchnic organs that is, the liver, gut, and kidneys (responsible for 20%–25% of total glucose disposal), and erythrocytes. The insulin dependent sites, namely the muscles, including the myocardium, and the adipose organ, account for the remaining 20%–25% of total disposal [10]. Thus, organ‐specific insulin‐resistance must be taken into consideration to ensure a thorough assessment of insulin‐resistance. Although studies have shown that increased hepatic glucose production, together with the inability to suppress it after meal ingestion, and increased lipolysis with free fatty acid release into the portal circulation are strongly implicated in whole‐body insulin resistance, uncertainty persists as to which defect plays the primary role in defining T2D [11].

These considerations underline the need to use different tools to assess insulin‐resistance in the different organs involved in regulating glucose metabolism to ensure that the contribution of each organ to the development of T2D is accurately understood.

Currently, glucose organ‐specific uptake can be investigated through positron emission tomography (PET) using radiotracers such as 2‐deoxy‐2‐[18F]fluoro‐D‐glucose ([18F]FDG). The latter is a glucose analogue that enters cells through glucose transporters via facilitated diffusion utilising the glucose gradient to cross the cell membrane. Unlike glucose, however, once inside the cell it is not metabolised. To identify the specific alteration in glucose metabolism [12], the functional data obtained by PET need to be integrated with anatomic images from computed tomography (CT) allowing us to visualise glucose uptake and its localisation [13]. A practical implication is that, over the years, [18F]FDG PET/CT has become a fundamental tool to detect cancer [14] since most malignant cancer cells increase their glucose uptake as well as their glycolytic rate in order to proliferate rapidly [15] in a process referred to as the Warburg Effect [16]. [18F]FDG PET accuracy may be affected by plasma glucose levels even though there is still no consensus as to how glycaemia affects standard uptake value, a measure of the relative uptake in a region of interest, in different organs and in conditions such as diabetes and insulin resistance [17].

The aim of this review article is to give an overview on how [18F]FDG can be used as a metabolic tracer in order to visualise organ‐specific glucose uptake (in muscle, liver, adipose organ, heart, brain, kidney) in different metabolic conditions such as insulin resistance and T2D.

## 2 Insulin Sensitivity Assessment

Over the years, several efforts have been made to develop comprehensive assessments of insulin sensitivity and resistance; however, challenges persist due to the lack of standardised methodologies and the high level of specialisation required.

The gold standard technique for assessing systemic insulin‐sensitivity is the hyperinsulinemic euglycemic clamp (HEC). By using 40 mIU/min of insulin adjusted for body surface (m^2^), first as a prime and then as a continuous infusion, a hyperinsulinemic state is achieved. After a few minutes, glucose is infused at a specified rate (GIR) to ‘clamp’ glucose plasma at fasting, steady‐state levels. The hyperinsulinemic state induces suppression of endogenous glucose production and promotes peripheral glucose disposal. If glucose is administered at variable infusion rates in order to achieve a steady euglycemic state, the amount infused equals glucose utilisation by all body tissues, thus quantifying the whole body glucose disposal [18, 19].

The alternative in vivo approaches should be time‐efficient, minimally invasive, and designed to limit the need for complex infusion setups or reliance on mathematical modelling.

Among the experimental techniques employed, the Insulin Tolerance Test [20] measures the rate at which exogenous insulin, given as a bolus, lowers blood glucose levels; the Frequently Sampled Intravenous Glucose Tolerance Test [21] applies a mathematical model to derive an insulin sensitivity index following an intravenous bolus of glucose; the Matsuda Index combines fasting and post‐load glucose and insulin values derived from the Oral Glucose Tolerance Test [22] and the Homoeostasis Model Assessment Insulin Resistance (HOMA‐IR index) [23] estimates insulin resistance from fasting glucose and insulin levels, reflecting primarily hepatic insulin resistance. Table 1 describes the main advantages and disadvantages of these approaches. HEC remains the best tool to evaluate whole body insulin‐sensitivity, but it mainly assesses muscle insulin‐sensitivity, is time consuming, and unfeasible in routine clinical practice. The overall effect of insulin is the sum of the combined dose‐response profiles of various organs, each responding differently to its action. Insulin receptors in the liver are generally the first to be maximally activated, followed by those in the adipose organ and finally in the muscle [25]. This means that in a hyperinsulinemic state, at higher insulin concentrations, the insulin receptors in both the liver and adipose organ are already saturated, thus allowing us to evaluate the response of the muscles.

**TABLE 1:** Experimental techniques employed to measure insulin sensitivity.

Insulin sensitivity assessment	Advantages	Disadvantages
Hyperinsulinemic euglycemic clamp [18]	Gold standard for assessing insulin‐sensitivity	Requires expertise and an appropriate setting with infusional pumps
Insulin tolerance test [24]	Simple to perform	Hypoglycemia
Frequently sampled intravenous glucose tolerance test [21]	Simple to perform	Hyperglycemia; requires mathematical modelling
Matsuda index [22]	Simple to perform OGTT still remains a validated tool for glucose tolerance assessment	Lower correlation with insulin‐sensitivity in case of beta cell dysfunction
Homoeostasis model assessment insulin resistance (HOMA‐IR index) [23]	Simple to perform	Mostly associated with hepatic insulin‐resistance

## 3 [18F]FDG PET

As stated above, glucose organ‐specific uptake and the qualitative aspects of insulin‐resistance can be investigated through PET, a functional imaging that detects positron‐emitting radiotracers. [18F]FDG is a glucose analogue used to perform an assessment of glucose metabolism. [18F]FDG is transported across cell membranes by glucose transporters (GLUTs) through a facilitated diffusion process. The two variables involved in defining glucose uptake are glucose concentration, forming a plasma‐cell gradient, and the GLUT density and conformational state on the plasmatic membrane, allowing for the quantification and visualisation of glucose entry into the cell [26]. Having crossed the membrane, in a similar way to glucose, [18F]FDG is then enzymatically phosphorylated by hexokinase into an intermediate, [18F]FDG‐6‐phosphate [27, 28]. Unlike glucose‐6‐phosphate, which then undergoes glycogenesis and glycolysis for the production of ATP, [18F]FDG‐6‐phosphate is retained in the cell for the duration of its radioactive half‐life (around 110 min). During this time, positrons are emitted by the radioactive decay of the radionuclide and collide with electrons, producing gamma rays (annihilation photons) and dynamic PET captures a series of images over time reflecting tracer distribution and uptake in tissues. By combining functional data provided by PET with anatomical images through computed tomography/magnetic resonance, we can trace glucose uptake in vivo [12, 13]. CT images provide high spatial resolution, whereas MR images are more suitable for soft tissue contrast and functional imaging capabilities (e.g., diffusion, perfusion).

For each region of interest (ROI), Time‐Activity Curves (TACs) are generated in order to show how tracer concentration varies over time. In order to compute metrics like glucose uptake rates, it is essential to use kinetic models to analyse tracer concentration in the blood over time. Dynamic models used to measure glucose uptake include two main approaches: compartmental models, which simulate the movement of FDG between plasma, tissue, and intracellular spaces, and simplified linear methods such as Patlak graphical analysis, which estimates net uptake by assuming FDG is irreversibly trapped within cells [29].

The time‐activity curve of plasma FDG is represented by the input function, which can be assessed in two ways. Although frequent arterial sampling is considered the gold standard, it is invasive and can be uncomfortable for subjects. A noninvasive method derives concentration curves from regions of interest in major arteries or cardiac chambers. However, the relative distance and blood dispersion between the heart and peripheral tissues introduce time delays that can distort results. Alternating chest and tissue visualisation can theoretically improve input function estimation. This approach may be practical for clinical and research settings but requires appropriate corrections for unintentional movements [30, 31]. To standardise results, it is essential to maintain a stable ambient temperature, since the latter can influence basal metabolic rate and tissue‐specific glucose uptake. In fact, temperature fluctuations can alter [^18^F]FDG distribution, especially in BAT, muscle, and skin [32]. In order to have the same reproducible environment, climate‐controlled rooms or the use of thermal blankets is beneficial (Table 2).

**TABLE 2:** Dynamic positron emission tomography.

1. Time activity curves	Tracer concentrations are analysed for every region of interest (ROI) through linear or compartment models.
2. Input function	Representing FDG input through invasive sampling or visualising the concentration in large vessels or cardiac chambers.
3. Standardisation of results	Standardised uptake values are produced
4. Temperature modulation or pharmacological stimuli (i.e. beta 3 agonists)	Dynamic tissue activity, such as that of brown adipose tissue, can be visualised using these methods.

A recent Swedish study has shown how whole‐body insulin resistance can be measured by conducting PET/magnetic resonance (MR) during a hyperinsulinemic euglycemic clamp, thus visualising glucose uptake [33]. The authors described how T2D subjects exhibited a significantly reduced glucose uptake in the muscle, adipose organ and liver, positively correlating with insulin sensitivity values, whereas glucose uptake in the brain was inversely correlated with it.

Building on these premises, we will explore glucose disposal and production in fasting and hyperinsulinemic conditions, and the consequent changes in glucose uptake during PET/FDG. We will begin with the primary tissues involved in glucose uptake (muscle and heart) and continue in order of importance from the different adipose organs to the liver and other organs.

Table 3 describes the main studies on organ‐specific insulin‐sensitivity assessment through PET.

**TABLE 3:** Organ‐specific insulin‐sensitivity.

Organ	Physiology	Insulin‐resistance/T2D	Reference study
Skeletal muscle	Insulin enhances FDG uptake in muscle by increasing glucose delivery, transport, and phosphorylation.	[18F]FDG uptake impairment during hyperinsulinemic euglycemic clamp.	Goodpaster [34]
Myocardium	Insulin significantly increases myocardial glucose uptake	[18F]FDG uptake impairment during hyperinsulinemic euglycemic clamp.	Hu [35]
Adipose	Insulin stimulates glucose uptake in both white and brown adipocytes. The BAT is metabolically more active with greater glucose uptake rates.	Visceral fat shows greater insulin resistance (reduced [18F]FDG uptake) than subcutaneous fat during hyperinsulinemic euglycemic clamp. Reduced blood flow/inflammation is a key determinant of adipose tissue insulin resistance.	Virtanen [36]
			Virtanen [37]
			Ferrannini [38]
			Goossens [39]
Liver	Both hyperinsulinemia and hyperglycemia stimulate liver glucose uptake through GLUT‐2.	Insulin‐mediated hepatic glucose uptake (reduced [18F]FDG uptake) is impaired in individuals with obesity and type 2 diabetes.	Iozzo [7]
			Iozzo [40]
Kidney	The renal cortex is an insulin‐sensitive tissue. Hyperinsulinemia increases glucose uptake in the cortical region.	Basal and insulin‐mediated [18F]FDG uptake in the medulla and cortex is reduced in obesity.	Rebelos [41]
Brain	Brain glucose uptake (BGU) through GLUT‐1 and GLUT‐3 reaches its peak under basal insulin conditions	In impaired glucose tolerance, [18F]FDG PET during hyperglycaemic euglycemic clamp does not increase brain glucose uptake.	Hirvonen [42]

## 4 Skeletal Muscle

As we know, in the resting state, the skeletal muscle relies on free fatty acid oxidation to produce most of the energy it needs, with a low glucose extraction rate compared to the brain. In hyperinsulinemia and physical exertion, muscle glucose utilisation can increase by 10 fold, accounting for the majority (∼85%) of glucose disposal [43]. On the one hand, hyperinsulinemia stimulates muscle vasodilatation, which results in increased blood flow, and on the other hand, GLUT‐4 translocation from intracellular vesicles to the plasma membrane allows increased muscular uptake [44, 45]. GLUT4 is largely expressed in insulin‐sensitive tissues (muscle and adipose organ) DeFronzo's ominous octet identifies skeletal muscle insulin resistance, resulting in reduced glucose uptake, as a core pathophysiological defect involved in hyperglycemia [7, 9]. Although PET studies are consistent with the evidence that muscle glucose uptake is reduced in obesity and type 2 diabetes [34], assessing the entity of muscle insulin resistance may be challenging due to the fibre heterogeneity, which includes both oxidative (type 1) and glycolytic (type 2) [46]. The role of [18F]FDG PET in unravelling glucose metabolism has been underlined in many studies. An interesting analysis has pointed out that FDG uptake is a more complex marker of glucose metabolism since it not only reflects glycolysis but also the consumption of glucose through cellular mechanisms [47].

In oxidative, type I–fibre–dominant muscle, higher levels of GLUT4s, insulin signalling proteins such as insulin receptor substrate (IRS) and mitochondria collectively account for increased glucose uptake, confirming the complexity of intracellular metabolism.

**FIGURE 1:**
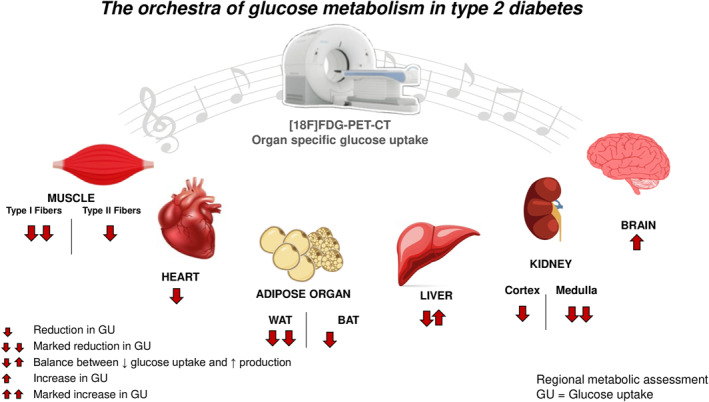
The orchestra of glucose metabolism in type 2 Diabetes: [18F]FDG PET‐CT provides a comprehensive insight into glucose metabolism enabling the identification of the various players involved in the *orchestra* of metabolism namely: muscle glucose uptake (GU) depends on the relative proportion of oxidative (type I fibres) to glycolytic muscle fibres (type II fibres); the heart exhibits reduced GU in insulin resistance conditions, especially in diabetes; the adipose organ shows a greater reduction in white adipose (WAT) glucose uptake compared to brown adipose tissue (BAT); the liver is central to glucose homoeostasis, facilitating hepatic GU, stimulated by high insulin and blood sugar. However, the hepatic first‐pass effect, where the liver clears a large portion (40%–80%) of pancreatic insulin, results in decreased insulin‐driven GU and, moreover, hepatic glucose production is increased. The kidney has a well‐defined structure in which the medulla shows higher GU rates compared to the cortex, resulting in lower FDG uptake in pathological conditions such as T2D and obesity; in the brain, an inverse correlation is seen between whole body insulin sensitivity and brain insulin‐sensitivity since cerebral tissue shows an enhanced glucose metabolism in T2D and obesity.

This concern was initially raised when performing HEC and administering 2‐[3H]deoxyglucose in animal models, which demonstrated that insulin promotes greater glucose uptake in muscles rich in oxidative fibres. These findings suggest that in vivo variations of insulin‐resistance from one individual to another may be due to the different relative proportions of oxidative to glycolytic muscle fibres (Figure 1). These results were confirmed in [18F]‐FDG studies suggesting that glucose transport is particularly impaired in specific skeletal muscle groups, probably due to muscle fibre type composition [48].

By combining a hyperinsulinemic euglycemic clamp with dynamic imaging of glucose uptake, it is possible to assess skeletal muscle insulin sensitivity without the influence of endogenous glucose production. However, this approach is often costly and laborious and may be ethically unfeasible. For this reason, researchers are attempting to create a surrogate index for skeletal muscle insulin sensitivity. For example, Klen et al., aimed to develop a predictive model to assess muscle insulin sensitivity through metabolomics in an attempt to integrate complex data. This predictive model is more efficient than the surrogate index Homa IR, which assesses insulin resistance from fasting glucose and insulinemia [49]. Indeed, these complex analyses are unfeasible in routine clinical practice, but they highlight the need to incorporate emerging knowledge.

Unfortunately, we still lack sufficient evidence to provide physicians with a better understanding of muscular insulin resistance and the prescription of personalised exercise regimens—whether aerobic or resistance training—targeting specific muscle groups. Moreover, as already mentioned, the muscle itself exhibits peculiar features due to its different fibres. Indeed, a specific surrogate index to assess targeted muscle insulin sensitivity, such as myocardial insulin sensitivity, would be useful in guiding clinical practice, verifying treatment benefit, and following up patients with specific metabolic conditions such as diabetes; however, this remains an unmet need.

## 5 Heart

The myocardium is metabolically flexible, and adapts to substrate and oxygen availability [50]. The Randle cycle describes the reciprocal relationship between fatty acid oxidation and glucose utilisation:

when FFAs are abundant, they are preferentially oxidised, suppressing glucose oxidation and viceversa [51]. Indeed, glucose typically contributes about 20% of the total energy supply, a proportion that becomes crucial in pathological conditions (i.e., ischaemia) or in anaerobic states. Under fasting and aerobic conditions, myocardial cells rely mostly on beta‐oxidation of free fatty acids. These considerations are the reason why the dietary regimen in the days prior to the PET evaluation is essential for reliable interpretation. If a suppression of physiological glucose uptake is required to highlight inflamed myocardium or valves, a high‐fat, low‐carbohydrate diet is necessary [52, 53]. On the other hand, in order to study cardiac glucose metabolism, hyperglycemia must be induced through intravenous infusion to reduce fatty acid consumption. Myocardial [18F]FDG uptake can be focal, regional or diffuse [54]. Positron emission tomography has been widely used to study myocardial glucose metabolism using [18F]‐FDG in different pathological settings, and also to assess tissue perfusion with [13N]‐ammonia [55]. A pivotal PET study by Iozzo and colleagues demonstrated that insulin is able to enhance myocardial glucose uptake (MGU) and myocardial blood flow (MBF) in healthy individuals, and can even redistribute blood flow to different areas of the heart [56]. Evidence suggests that cardiovascular risk factors involved in metabolic syndrome and in the spectrum of altered glucose tolerance that precedes diabetes are associated with reduced myocardial glucose consumption [57] and reduced metabolic flexibility, thus compromising the myocardium under hypoxic conditions. PET/CT scan images, in fact, yield significantly lower myocardial standard uptake values in type 2 diabetes subjects, as compared to impaired fasting glucose (IFG) and normal fasting glucose (NFG) subjects [35] (Figure 1). The analysis of myocardial glucose uptake with [18F]FDG PET/CT can thus provide important information to, for example, assess the effect of a therapy in modifying myocardial insulin resistance. We will discuss this specific topic later in this review, in the section ‘metabolic effects under pharmacological treatment’.

## 6 Adipose Organ

Studies conducted in the early 2000s have shed light on adipocyte glucose uptake in resting and insulin‐stimulated conditions both in obese and type 2 diabetes subjects [36, 48, 58], showing that both the subcutaneous and visceral adipose organs exhibit a lower glucose uptake rate in these conditions compared to metabolically healthy lean individuals. Over the years, several studies have been performed, yielding heterogeneous results and causing Ferrannini and colleagues [38] to speculate that in in vivo models of insulin‐resistance, a decreased regional blood flow to the adipose organ, both subcutaneous and visceral, may be involved in the impairment of insulin‐mediated glucose uptake. In their PET analysis, blood flow to the insulin‐resistant skeletal muscle is preserved, suggesting that a change in adipose organ physiology may be implicated. These findings support the potential of PET to identify core defects in muscle and adipose organ insulin‐resistance.

The adipose organ is composed of two morphologically/physiologically different tissues: white adipose tissue (WAT), found mainly in subcutaneous depots, and brown adipose tissue (BAT), which is mainly visceral, and a more vascularised and metabolically active depot compared to WAT [59, 60] Indeed, FGD/PET has also clarified that not only is BAT metabolically active (with greater glucose uptake than WAT) but that, although it is mainly expressed during childhood, it also persists into adulthood [37, 59, 61, 62] The main activity of BAT tissue is to utilise glucose from the circulation, especially after cold exposure or exercise, in order to induce an adaptive thermogenesis. In fact, in adults BAT is located in the visceral adipose tissue depots around the mediastinum vascular and abdominal vascular trunks (aorta and its branches) in order to rapidly distribute the heat produced to the rest of the body [63].

Studies have shown that BAT volume is inversely associated with whole‐body adiposity, suggesting a possible role in the protection against obesity [64]. In insulin‐resistant states or during prolonged fasting, BAT glucose uptake is reduced [65], suggesting a decreased activity of the tissue (Figure 1).

By permitting the visualisation of the metabolic improvements associated with increased BAT activity, PET studies have also shed light on the capacity, known as *browning,* of white adipose tissue to transdifferentiate into brown adipose tissue [66, 67, 68]. In this context, beige (brite) adipocytes are the morphological feature of the transdifferentiation of white into brown adipose tissue [63] and share common characteristics with white and brown adipose tissue. They emerge within white fat depots in response to physiologic stimuli like cold exposure or β3‐adrenergic activation [69] or pharmacologic agents (e.g., mirabegron [70], tirzepatide [71], dapagliflozin [72]). [^18^F]FDG PET provides a valuable tool to dynamically assess glucose uptake in thermogenic fat, allowing precise quantification of its metabolic activation. Several studies have demonstrated a significant improvement in insulin resistance after increased brown adipose tissue activity [73, 74, 75]. On the other hand, *whitening* of brown adipose tissue (i.e., transdifferentiation of brown adipocyte into white adipocyte) can occur in dysmetabolic conditions such as obesity and T2D [76, 77] This phenomenon leads to increased adipocyte death, as brown adipocytes have a lower critical size for cell death compared to white adipocytes. This is associated with low‐grade inflammation triggered by macrophages in response to adipocyte death, which exacerbates and contributes to insulin resistance [76, 78, 79, 80]. Under insulin stimulation, visceral adiposity shows higher glucose‐uptake rates than subcutaneous adipose tissue, possibly due to a differential metabolic activity (more BAT), as Christen and colleagues suggest [81]. The reduction of visceral FDG uptake may thus indicate a reduction of visceral BAT activity and, indirectly, a whitening of the tissue (Figure 1).

Epicardial adipose tissue (EAT), which is a visceral fat depot located between the myocardium and the visceral layer of the pericardium, has recently garnered increasing attention [82]. Due to its unique characteristics, it provides both structural and mechanical protection; however, due to its pro‐inflammatory profile, particularly in conditions like T2D, it also contributes to the development of cardiovascular diseases. Epicardial adipose tissue can be assessed through PET imaging [83].

FDG‐PET has also been used to detect changes in bone marrow glucose metabolism, which may reflect bone marrow adipocyte activity, haematopoietic function, or disease states [84].

To summarise, FDG‐PET is a valuable tool for investigating the metabolic activity of adipose organs in both healthy and pathological conditions. It provides insight into restoring metabolic function and identifies new targets to address diseases such as obesity and T2D [60] The role of adipose organs depends on the balance between white and brown fat tissue, with each type contributing to insulin resistance in both positive and negative ways, based on their distinct characteristics (Figure 1).

## 7 Liver

In a fasted state, the body relies on endogenous glucose production, primarily hepatic glucose production (HGP), to meet its energy needs. The liver is a key organ in glucose homoeostasis allowing hepatic glucose uptake (HGU) through GLUT‐2 [85], which is stimulated by both hyperinsulinemia and hyperglycaemia [86]. Due to the hepatic first‐pass effect, 40%–80% of insulin released by the pancreas is cleared [86], leading to reduced insulin‐stimulated hepatic glucose uptake [87]. This feature makes the tracing of glucose in the liver, and therefore the study of the hepatic glucose uptake (HGU) more difficult. PET represents a valuable tool to investigate hepatic glucose metabolism, since invasive arterio‐venous gradient sampling is not feasible for evaluating HGU and HGP [88]. One of the limitations of PET in this context is that the liver has a double circulation, receiving its blood supply from the venous portal system (80%) and the hepatic artery (20%): when administering the radiotracer, this is first distributed to the splanchnic organs and its concentration dispersed due to metabolic processes [88], preventing a precise tracing of glucose destiny. Because of this dual‐input model, the hepatic FDG time–activity curve differs from other organs that receive a single (arterial) input [89].

To correct for the underestimation of hepatic FDG uptake observed in single‐input models, some authors have proposed mathematical models that account for portal input by incorporating gut activity curves as well as transit delays and dispersion before the tracer reaches the liver.

The Patlak model was introduced to simplify kinetic analysis by estimating the net influx rate (Ki) of FDG into the tissue and tracking FDG concentration in tissue and plasma over time. Moreover, recent improvements in PET scanner technology can improve the accuracy of dual‐input kinetic modelling [86].

It is well known that insulin‐mediated hepatic glucose uptake (HGU) is impaired in individuals with obesity and type 2 diabetes [7, 40]. In contrast with other tissues, the liver expresses a dephosphorylation enzyme, which allows glucose‐6‐phospate to be converted into glucose once again. In a recent study [86], the authors analysed FDG kinetics using constant rate parameters—blood clearance (k1), phosphorylation (k2), dephosphorylation (k3), and reappearance in the blood (k4)—to model the process. Their findings show that the increased activity of glucose‐6‐phosphatase, driven by the failure of insulin to suppress dephosphorylation, ultimately leads to elevated hepatic glucose production (HGP) (Figure 1). Moreover, [18F]‐FDG provides quantitative metabolic imaging, allowing the detection of metabolic dysfunction‐associated fatty liver disease (MASLD) and assessment of early fibrosis using a non‐invasive method [90]. Additionally, the use of 18F‐labelled fatty acid analogue allows the assessment of hepatic fatty acid uptake [91].

These findings highlight the potential of PET imaging to identify key steps in the metabolic disturbances that are characteristic of type 2 diabetes and insulin resistance, particularly those related to altered gluconeogenesis and glycogen synthesis. However, its application currently remains limited to research settings and is not yet suitable for routine clinical use.

## 8 Kidney

The renal parenchyma is a metabolically active tissue. After glomerular filtration, glucose is reabsorbed in the convoluted proximal tubule through the action of SGLT‐1 and SGLT‐2. The metabolic demands of the kidneys are primarily met by local gluconeogenesis [92]. The use of PET to visually analyse renal glucose metabolism has until now been limited due to the complex structure of the organ comprising a cortical, medullary and pelvic region. Administered [18F]FDG is filtered by the glomerulus but not entirely reabsorbed by SGLT‐1 and SGLT‐2 [93] due to its low affinity, leading to a partial accumulation in the tubular region [41]. In recent years, Rebelos and colleagues have used [18F]‐FDG PET to show how the kidney acts as an insulin‐sensitive tissue in a fasting and post‐prandial state (by inducing hyperinsulinemia), thus providing new evidence [41]. Comparing lean and obese subjects, after adjusting the results for the residual intratubular [18F]FDG, the insulin‐resistant subjects exhibited lower glucose uptake in both the medulla and cortex on [18F]FDG‐PET scans [94], both during fasting and insulin‐stimulated conditions. More specifically, under fasting conditions, the medulla exhibited a higher glucose uptake rate, suggesting increased glucose consumption to support oxidative metabolism (Figure 1). In contrast, the cortex appeared to rely on different substrates and was more involved in gluconeogenesis. These dynamic imaging data confirm previous findings in animal models [95]. In fact, as Mather and Pollock have shown, the net organ balance of glucose is around zero, reflecting the difference between renal glucose release by the cortex and renal glucose uptake by the medulla. Additional findings from PET studies suggest that hyperinsulinemia induces an increase in cortical [18F]FDG uptake compared with the medulla, confirming the localisation of insulin‐receptors on the basolateral side of tubuli [41]. In T2D, augmented renal glucose reabsorption, by a threshold increase, is involved in defining hyperglycemia [9] and enhanced renal gluconeogenesis may be responsible for the accumulation of glycogen in the kidneys [96].

Thus, the kidney has a heterogenous structure with different metabolic pathways and plays an active role not only in filtering but also in reabsorbing glucose. We can therefore speculate that in vivo imaging of renal metabolism may help better understand its role in conditions where gluconeogenesis might be impaired (i.e., liver failure) or its counterregulatory response to hypoglycemia, which seems to be impaired in patients with type 1 diabetes [97].

## 9 Brain

In the basal state (after an overnight 10–14 hour fast) glucose is spared in order to ‘feed’ the brain, which relies on glucose as its fundamental fuel and on ketones as an alternative substrate [98].

Although the brain's share of total uptake is large (−50%), the amount of glucose cleared per plasma volume (fractional uptake rate) is about 9%, reflecting the efficiency of glucose extraction [99].

GLUT‐1 and GLUT‐3 are the insulin‐independent glucose transporters involved in cerebral metabolism [100]. PET imaging studies show that the brain is responsible for at least 50% of whole‐body glucose disposal during the fasting state and 10%–20% during euglycemic insulin stimulation [101].

Early studies have indicated that, unlike other tissues such as the liver and the muscle, brain glucose uptake (BGU) in healthy individuals reaches its peak under basal insulin conditions, meaning that insulin stimulation does not induce further effects [102]. However, brain hypermetabolism with an increase in BGU has been demonstrated in obesity [100, 103] and impaired glucose tolerance [42] (Figure 1). Although it is unknown whether insulin‐resistance directly affects [18F]FDG cerebral uptake, the correlation between increased brain glucose uptake and whole‐body insulin resistance in type 2 diabetes subjects may reflect a central/autonomic alteration in glucose metabolism [104]. Moreover, new evidence has shown that T2D subjects with cognitive impairment exhibit a reduction in glucose uptake in specific brain regions [105] such as the frontal, temporal‐parietal lobes, cingulate gyrus, and hippocampus, that is, key structures associated with higher cortical functions. Although neuronal glucose uptake primarily relies on insulin‐independent GLUT1 and GLUT3 transporters, impaired insulin signalling affects endothelial function by reducing nitric oxide release and neuronal glucose utilisation, leading to decreased cerebral glucose uptake and regional hypometabolism [100, 106, 107].

In a recent [18F]FDG PET analysis, the authors discovered that late middle‐aged adults with higher HOMA‐IR exhibited reduced cerebral glucose metabolism, and this decrease in glucose metabolism was associated with poorer memory function [108] Moreover, a correlation has been found between APOE‐ε4 genotype and altered glucose cerebral metabolism, although the mechanisms are still unclear [108, 109]. Since [18F]‐FDG‐PET can help detect early metabolic changes before structural damage occurs [110], it may represent an important tool in identifying individuals at risk of cognitive decline or neurodegeneration due to insulin resistance or diabetes.

## 10 Visualising Metabolic Effects Under Pharmacological Treatment

Several drugs for the treatment of type 2 diabetes, such as metformin and thiazolidinedione, have demonstrated effects on metabolism [111]. However, the newest glucose lowering drugs, including glucagon‐like peptide 1 receptor agonists (GLP‐1RA) and sodium‐glucose cotransporter 2 inhibitors (SGLT‐2i), have changed the diktat ‘*treat to target*’ into ‘*treat to benefit*’. Indeed, their effects go beyond lowering blood glucose levels, and recent evidence has shown systemic improvements in patients treated with these drugs, particularly in terms of cardiovascular and renal protection [112].

Functional studies with both SGLT‐2i and GLP‐1RA have shown metabolic changes in the diabetic heart. In our recent DapaHeart trial, PET‐CT scan evaluations performed during euglycemic‐hyperinsulinemic clamps (HEC) revealed that even a short‐term treatment (4 weeks) with the SGLT‐2i dapagliflozin induced a 30% increase in myocardial flow reserve (MFR) compared to placebo in patients with stable coronary artery disease [113]. Moreover, we found that treatment was associated with a reduction in epicardial adipose tissue (EAT) thickness and FDG uptake [114]. The increase in MFR and the reduction in EAT were comparable, suggesting a potential link between the two parameters. This raises the possibility that decreased EAT thickness may lead to reduced EAT inflammation—evidenced by lower FDG uptake—which in turn could contribute to improved microvascular function and MFR. Moreover, the anti‐inflammatory effects of SGLT‐2i have been ascribed to their particular mechanism of action, which lowers glucose by promoting its elimination through urine, thus inducing a net loss of calories. This effect promotes a metabolic shift at the systemic level, promoting ketones and fatty acids as alternative substrates to glucose. This, in turn, modulates the major nutrient‐sensing pathways fuelling chronic low‐grade inflammation, for example, mTOR and the inflammasome, mimicking major features of caloric restriction [115, 116, 117] Indeed, Solis‐Herrera and colleagues have shown that incremental infusions of ketone beta‐hydroxy‐butyrate (β‐OH‐B) cause a dose‐response increase in left ventricular ejection fraction and myocardial blood flow, providing another potential explanation for the benefits induced by SLGT‐2i [118].

Reduction in cardiovascular events associated with GLP‐1 receptor agonists (GLP1‐RAs) has also been described [119] and may be attributed to decreased vascular inflammation. Studies with animal models have shown that treatment with the GLP‐1RA semaglutide decreases vascular uptake of [18F]FDG, supporting the hypothesis that GLP1‐RA treatment reduces vascular inflammation [120]. However, these results have not been confirmed in humans with type 2 diabetes, as no changes in vascular inflammation, as assessed by [18F]‐FDG uptake, were observed [121]. On the other hand, GLP1‐RA treatment has shown neuroprotective benefits in animal and human models [122]. A recent systemic review concludes that GLP‐1RA treatment is not associated with a reduction in amyloid deposition or neurofibrillary tangles, but speculates that the potential cognitive improvements observed in large trials may be attributed to vascular effects [123]. We can speculate that the shared mechanism underlying the cardiovascular and renal protection offered by both SGLT‐2 inhibitors and GLP‐1 receptor agonists is the reduction of visceral fat in various locations. This, in turn, may decrease the pro‐inflammatory state, as outlined above, and improve insulin sensitivity. Moreover, both SGLT‐2i and incretin analogues have some WAT browning properties [117, 124, 125], which can, in part, justify the improvements in insulin sensitivity observed with these treatments.

Briefly, as mentioned above, metformin and thiazolidinedione are also drugs with a metabolic impact. In a randomised trial, Viljanen and colleagues [126] used a PET evaluation to explore the impact of the thiazolidinedione rosiglitazone on subcutaneous adipose tissue glucose uptake and blood flow compared to metformin. The group treated with rosiglitazone showed an increase in subcutaneous glucose uptake along with an enhanced regional perfusion, suggesting that the increased adipose tissue insulin sensitivity, already described in humans after rosiglitazone treatment, could be mediated by the increased tissue perfusion. On the other hand, with metformin, they saw a decrease in hepatic glucose production. As a confirmation of our previous observations, FDG uptake does not perfectly reflect glycolytic flux, as it captures only glucose entry and trapping rather than its complete consumption. Metformin inhibits the first enzyme in the mitochondrial electron transport chain (respiratory complex I), thereby reducing oxidative phosphorylation, and lowering mitochondrial ATP production. As a positive effect, glycolysis is upregulated to meet energy demands, ultimately increasing glucose consumption. Therefore, the visualisation of FDG uptake during a pharmacological treatment with metformin may vary across tissues, such as the adipose organ, muscle, and intestine [127]. Moreover, metformin promotes lactate production by any tissue and cell containing glycogen and/or taking up glucose, which can be directly oxidised for immediate use bypassing glycolysis [128]. The possibility of quantifying (and visualising) metabolic changes after specific treatment represents a fundamental tool to provide new insight not only on the effect of new therapies but also on the pathogenesis of the diseases, hypothetically providing new targets to treat them.

## 11 Conclusions

Type 2 diabetes is a complex disease characterised by the interplay of multiple pathophysiological defects. Insulin resistance has long been considered as being primarily muscular, and metabolic assessments, such as HEC, present limitations in evaluating whole body insulin sensitivity. Emerging evidence highlights that the organs discussed in this review contribute to systemic insulin resistance in ways that differ both qualitatively and quantitatively. Dynamic imaging acquisition through PET, using appropriate radiotracers, is a powerful tool for studying regional glucose metabolism, providing valuable insight into how the different organs are affected by metabolic alterations. Over the past 20 years, significant advancements have been made; however, adequate training is required especially in the use of metabolic dynamic tests to create specific conditions. PET studies do indeed require substantial economic resources, and routine clinical use may not be feasible due to exposure to CT radiation.

[18F]FDG PET provides a comprehensive insight into glucose metabolism enabling the identification of the different players involved in the *orchestra* of metabolism (Figure 1). Every single organ plays a specific quantitative and qualitative role in defining whole body insulin‐resistance, which differs from subject to subject. The identification of the specific alterations involved paves the way for the development of new therapeutic and personalised strategies, especially with the implementation of new tissue‐specific radiotracers.

## Author Contributions

Shawn Gugliandolo, Andrea Giaccari and Francesca Cinti wrote the main manuscript. Cassandra Morciano, Lucia Leccisotti, Umberto Capece, Gianfranco Di Giuseppe, Teresa Mezza, Gea Ciccarelli, Laura Soldovieri, Michela Brunetti, Adriana Avolio, Amelia Splendore and Alfredo Pontecorvi wrote parts of the manuscript. Shawn Gugliandolo, Cassandra Morciano and Francesca Cinti prepared the graphical abstract. Shawn Gugliandolo, Andrea Giaccari and Francesca Cinti edited and formatted the manuscript. All authors reviewed the manuscript.

## Conflicts of Interest

The authors declare no conflicts of interest..

## Data Availability

Data sharing not applicable to this article as no datasets were generated or analysed during the current study.
